# Development of an algorithm to aid triage decisions for intensive care unit admission: a clinical vignette and retrospective cohort study

**DOI:** 10.1186/s13054-016-1262-0

**Published:** 2016-04-02

**Authors:** Joao Gabriel Rosa Ramos, Beatriz Perondi, Roger Daglius Dias, Leandro Costa Miranda, Claudio Cohen, Carlos Roberto Ribeiro Carvalho, Irineu Tadeu Velasco, Daniel Neves Forte

**Affiliations:** Medical sciences doctoral program, University of Sao Paulo Medical School, Sao Paulo, Brazil; Intensive Care Unit, Hospital Sao Rafael, Salvador, Brazil; UNIME Medical School, Lauro de Freitas, Brazil; Emergency Department, Hospital das Clinicas, University of Sao Paulo Medical School, Sao Paulo, Brazil; Intensive Care Unit, Hospital Nove de Julho, Sao Paulo, Brazil; Bioethics Committee, Hospital das Clinicas, University of Sao Paulo Medical School, Sao Paulo, Brazil; Discipline of Bioethics, University of Sao Paulo Medical School, Sao Paulo, Brazil; Pulmonary Division, Heart Institute (InCor), Hospital das Clinicas, University of Sao Paulo Medical School, Sao Paulo, Brazil; Intensive Care Unit, Emergency Medicine Discipline, Hospital das Clinicas, University of Sao Paulo Medical School, Sao Paulo, Brazil; Palliative Care Team, Hospital Sirio-Libanes, Sao Paulo, Brazil

## Abstract

**Background:**

Intensive care unit (ICU) admission triage is performed routinely and is often based solely on clinical judgment, which could mask biases. A computerized algorithm to aid ICU triage decisions was developed to classify patients into the Society of Critical Care Medicine’s prioritization system. In this study, we sought to evaluate the reliability and validity of this algorithm.

**Methods:**

Nine senior physicians evaluated forty clinical vignettes based on real patients. The reference standard was defined as the priorities ascribed by two investigators with full access to patients’ records. Agreement of algorithm-based priorities with the reference standard and with intuitive priorities provided by the physicians were evaluated. Correlations between algorithm prioritization and physicians’ judgment of the appropriateness of ICU admissions in scarcity and nonscarcity settings were also evaluated. Validity was further assessed by retrospectively applying this algorithm to 603 patients with requests for ICU admission for association with clinical outcomes.

**Results:**

Agreement between algorithm-based priorities and the reference standard was substantial, with a median κ of 0.72 (interquartile range [IQR] 0.52–0.77). Algorithm-based priorities demonstrated higher interrater reliability (overall κ 0.61, 95 % confidence interval [CI] 0.57–0.65; median percentage agreement 0.64, IQR 0.59–0.70) than physicians’ intuitive prioritization (overall κ 0.51, 95 % CI 0.47–0.55; median percentage agreement 0.49, IQR 0.44–0.56) (*p* = 0.001). Algorithm-based priorities were also associated with physicians’ judgment of appropriateness of ICU admission (priorities 1, 2, 3, and 4 vignettes would be admitted to the last ICU bed in 83.7 %, 61.2 %, 45.2 %, and 16.8 % of the scenarios, respectively; *p* < 0.001) and with actual ICU admission, palliative care consultation, and hospital mortality in the retrospective cohort.

**Conclusions:**

This ICU admission triage algorithm demonstrated good reliability and validity. However, more studies are needed to evaluate a difference in benefit of ICU admission justifying the admission of one priority stratum over the others.

**Electronic supplementary material:**

The online version of this article (doi:10.1186/s13054-016-1262-0) contains supplementary material, which is available to authorized users.

## Background

Intensive care unit (ICU) admission triage is performed routinely in ICUs worldwide [1, 2], and patients who are refused ICU admission may have a higher risk of death [2]. This triage process is associated with several factors, including clinical characteristics of the patients [2–4], but it is also influenced by nonclinical factors [2, 4, 5]. Moreover, it has been shown that physicians may not follow triage recommendations [3], and concern has been raised that these clinical judgments could mask prejudice or bias [1]. So, development of objective directives and guidelines applying to individual patients, along with the need for an objective triage score, has been discussed in the literature [6, 7].

The Society of Critical Care Medicine (SCCM) has established guidelines for ICU triage [8], which categorizes patients into four priority strata. Priority 1 patients usually have no therapeutic limits and are critically ill unstable patients in need of intensive treatment and monitoring that cannot be provided outside the ICU. Priority 2 patients usually have no therapeutic limits, require intensive monitoring, and may potentially need immediate intervention. Priority 3 patients are critically ill but have a reduced likelihood of recovery because of underlying disease or the nature of their acute illness; these patients may have therapeutic limitations. Priority 4 patients are generally not appropriate for ICU admission, because either (1) they have little or no anticipated benefit from ICU care based on low risk of active intervention that could not safely be administered in a non-ICU setting or (2) they are patients with terminal and irreversible illness and face imminent death. However, these guidelines have not been formally evaluated.

There is no established gold standard for appropriateness of ICU triage, so, to assess the usefulness of any tool aimed at improving this process, it is important to use more than one method of evaluation and to demonstrate the reliability and validity of the tool [9, 10]. *Reliability* “refers to the degree to which repeated assessments of the same patient with a triage instrument will deliver the same acuity level,” as such yielding reproducible results [9, 11]. *Validity* would refer “to the degree with which the measured acuity level reflects the patient’s true acuity at the time of triage” [9], or the capacity of an instrument to reflect what it is proposed to measure. Validity is further delineated into three major types: content (i.e., criteria fit with current knowledge), construct (criteria measure what they are supposed to measure), and criterion (new criteria agree with existing standard) [10, 11].

To help in triage decision making for ICU admission in a setting of ICU bed scarcity, the Hospital das Clinicas da Faculdade de Medicina de Sao Paulo (HCFMUSP) assembled a critical care team to come up with a set of defined criteria for ICU admission. This group proposed an algorithm to aid triage decision making based on the SCCM’s guidelines. The algorithm was designed to help standardize, not replace, clinical judgment in ICU triage. We sought to evaluate the reliability and validity of this algorithm.

## Methods

### Ethics, consent, and permission

This single-center study was approved, and a waiver for informed consent was granted, by the HCFMUSP Research Ethics Committee (approval number 638.864).

### Setting

Sao Paulo is the largest city in South America, with an estimated population of 11 million. HCFMUSP is the largest hospital complex in South America, with about 2200 hospital beds. The Central Institute is the main building of the complex, an academic tertiary hospital with about 1100 hospital beds and 110 ICU beds divided among 10 ICUs. Despite the large number of ICU beds, there is a perceived shortage of ICU beds in the HCFMUSP complex due to the fact that it is a referral center for complex patients from the entire Sao Paulo state.

### Algorithm development

A team comprised of critical care specialists, bioethicists, and hospital managers was assembled to come up with an instrument to help triage decisions for ICU admissions. Content (logical) validity was achieved, and the final form of the algorithm was developed through a series of consensus meetings. The algorithm was approved by the hospital’s board and was based on the responses to four closed questions.

Question 1 asked whether the ICU request was for active intervention or monitoring. *Intervention* was defined as the need for vasoactive drugs, mechanical ventilation (invasive or noninvasive), or urgent hemodialysis in unstable patients. *Monitoring* was defined as the need for active monitoring with possibility of active intervention (e.g., high-risk surgical patients, acute coronary syndromes, postthrombolysis stroke patients).

Question 2 asked about patients’ comorbidities. Comorbidities were classified into four strata: (1) no comorbidities, (2) compensated comorbidities, (3) decompensated comorbidities (frequent hospital admissions in the last few months, unintended weight loss or loss of functionality), and (4) advanced disease with a probable life expectancy of months (metastatic cancer or locally invasive cancer, (advanced heart failure – i.e., American College of Cardiology/American Heart Association stage “D”) chronic obstructive pulmonary disease with hypoxemia and dyspnea at rest without relief with bronchodilators, National Kidney Foundation Kidney Disease Outcomes Quality Initiative chronic kidney disease stage 5 with contraindications to hemodialysis, Child-Pugh class C cirrhosis with contraindications to liver transplant, dementia with total loss of functionality and/or frailty syndrome and/or immobility syndrome, pressure ulcers, malnutrition, loss of sphincter control). These definitions were adapted from hospice indications published by the National Academy of Palliative Care [12].

Question 3 asked about patient’s previous functionality, as defined by activities of daily living (ADL) according to Katz and colleagues [13]. Patients were classified as functionally independent, partially dependent, or severely dependent (capable of performing a maximum of two ADLs).

Question 4 asked about the requesting physician’s most probable intuitive prognosis. Patients were classified as probable survivors without severe disabilities, probable survivors with severe disabilities, or probable nonsurvivors.

### Algorithm description

According to the responses to the four closed questions described above, a Microsoft Access 2013® Visual Basic for Applications (Microsoft, Redmond, WA, USA) algorithm was developed to classify patients into one of the four SCCM priority classes. Patients would be ascribed priority 4 if they had decompensated comorbidities with severe dependency, advanced disease with partial or severe dependency, or advanced disease with preserved functionality but an estimated intuitive prognosis of death. Patients were ascribed priority 3 if they had compensated comorbidities but severe dependency, decompensated comorbidities with partial dependence, or advanced disease with preserved functionality and an estimated prognosis of survival. All other patients were ascribed priority 1 or 2 on the basis of whether the ICU admission request was for active intervention or monitoring. The algorithm’s framework is available in Additional file 1.

### Study design

An invited convenience sample of 10 senior physicians with experience in emergency medicine or critical care evaluated 40 clinical vignettes developed with information retrieved from representative real patients for whom urgent ICU admission was requested at the HCFMUSP in January 2014. Case vignettes rather than actual charts were chosen to ensure that only information that was available at the moment of ICU request would be presented to the responding physicians.

These vignettes contained real information available at the moment of the patient’s request for ICU admission, such as age, sex, length of hospitalization, comorbidities, previous functional status, acute diagnosis, presence of organ dysfunction, need for advanced life support, and objective reason for ICU admission request. Sample vignettes are available in Additional file 1.

The physicians were asked to ascribe an intuitive prioritization of 1–4 following SCCM guidelines (physician-based priority). In addition, on the basis of information provided with each vignette, the physicians would answer the four closed questions of the algorithm, and then, according to the responses, the algorithm would ascribe a priority (algorithm-based priority). Physicians were blinded to this algorithm classification because it was performed offline.

Moreover, physicians were asked for their clinical judgment of the appropriateness of ICU admission for each clinical vignette. Appropriateness of ICU admission was evaluated in a non-ICU bed scarcity setting using a Likert scale with four levels (1 = completely agree with ICU admission, 2 = agree, 3 = disagree, and 4 = completely disagree). This Likert scale was further dichotomized into two strata (1 or 2 = appropriate admission and 3 or 4 = inappropriate admission). Appropriateness of ICU admission was also evaluated in an ICU bed scarcity setting by asking if the physician would admit the patient in each clinical vignette to the last ICU bed (yes-or-no question).

### Reference standard

These 40 clinical vignettes were also classified into the SCCM priority categories by subjective consensus between the two investigators (DNF and JGRR) who had access to the patients’ complete medical records, including outcomes (reference standard). These investigators were blinded to the physician-based and algorithm-based priorities. According to this reference standard, 7 cases reflected priority 1, 13 reflected priority 2, 10 reflected priority 3, and 10 reflected priority 4.

### Construct validity analysis

Construct validity is “the degree to which a test measures what it claims, or purport, to be measuring” [9, 11]. In this study, construct validity was determined by assessing how the algorithm would correlate to experts’ prioritization. It was evaluated by comparing priorities ascribed by the algorithm, based on physician’s responses (algorithm-based priority), to priorities ascribed by the two investigators with full access to patients’ medical records (reference standard), and to intuitive priorities ascribed by the physicians who evaluated the vignettes (physician-based priority). It was further assessed by evaluating the correlation of algorithm-based priorities with the clinical judgment of appropriateness of ICU admission in ICU scarcity or nonscarcity settings.

### Reliability analysis

Interrater reliability was evaluated by overall agreement among pairwise algorithm-based priorities and among pairwise physician-based priorities. Moreover, to assess the impact of each individual component of the algorithm, interrater agreement among physicians’ responses to each of the four individual questions was evaluated.

### Criterion validity analysis

Criterion validity is the extent to which a measure is related to an outcome [9, 11]. Because there is no gold standard for ICU triage, criterion validity was assessed by evaluating the predictive validity of the algorithm (i.e., by comparison with later outcomes believed to be associated with ICU triage) [9, 11].

Criterion validity was evaluated by applying the algorithm to a sample of patients with urgent ICU admission requests between September and December 2013 to assess the association of algorithm-based priorities with clinical outcomes. This sample was retrospectively retrieved from a database with administrative information and responses to the algorithm.

Importantly, the four questions of the algorithm were responded to prospectively at the moment of ICU request by the attending physician, but with the sole intent of evaluating the feasibility of the process. The responses to the questions and the priority ascribed by the algorithm were maintained offline and were not available to the triaging physicians, so they had no impact on actual ICU triage decisions. Patients younger than 16 years of age, repeated ICU requests, and requests for elective surgeries were excluded from the analysis.

We evaluated the association of algorithm-based priorities with hospital mortality, palliative care consultation, and ICU admission. The association of each of the four questions from the ICU request form individually with hospital mortality and ICU admission was also evaluated.

### Statistical analysis

Microsoft Excel 2013® (Microsoft) and Microsoft Access 2013® (Microsoft) were used as database software. Statistical analyses were performed with SPSS 13.0™ (SPSS Inc., Chicago, IL, USA) or Epi Info 7™ for Windows (Centers for Disease Control and Prevention, Atlanta, GA, USA). κ-statistic quadratic weighted analyses were performed using the VassarStats website [14].

Estimating a 50 % refusal rate with final values *n* = 5, α = 0.05, β = 0.20, ρ_0_ = 0.4, and ρ_1_ = 0.6, the sample size of observations was estimated as 35 [15]. The classification model designed by Landis and Koch [16] was used for the interpretation of κ. For the clinical outcomes analysis, based on previous findings [17] and preliminary analysis of our data (data not shown), we estimated that, to obtain a power of 80 % and a two-sided confidence level of 95 %, a sample size of 341 patients would be necessary to find an estimated odds ratio for mortality of 2.0, comparing lower priorities with higher priorities, estimating a hospital mortality rate of 30 % in the higher priorities group.

Reliability was evaluated by quadratic weighted κ, proportions (percentage) of agreement, and intraclass correlation coefficient. Categorical variables were described as numbers of cases and proportions. Continuous variables were described as mean ± standard deviation or median (interquartile range [IQR]). Differences in proportions were evaluated with χ^2^ statistics or Fisher’s exact test as appropriate. Differences in means and medians were evaluated with analysis of variance or the Mann-Whitney *U* test. A two-tailed *p* value less than 0.05 was considered statistically significant.

## Results

### Clinical vignette data

Of 10 physicians invited, 9 (90 %) returned the questionnaire, comprising 36 individual pairs of agreement. All physicians evaluated 40 clinical vignettes, completing 360 triage scenarios. There was incomplete data for 3 triage scenarios, so 357 triage scenarios were evaluated.

### Reference standard

Agreement between algorithm-based prioritization and the reference standard was substantial, with a median κ of 0.72 (IQR 0.52–0.77), which was not statistically different from median κ between physician-based priorities and the reference standard, which was 0.62 (IQR 0.57–0.70) (*p* = 0.258). Agreement between each physician-based priorities and algorithm-based priorities resulted in a median κ of 0.63 (IQR 0.55–0.74).

### Correlation with appropriateness of ICU admission

**Fig. 1:**
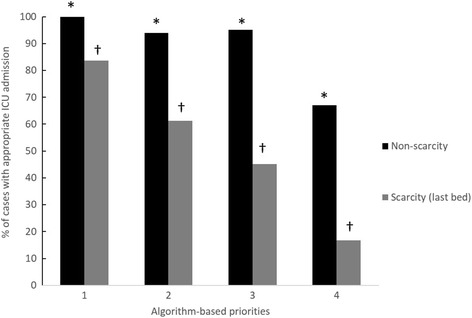
Proportion of scenarios in which patients would be admitted to the intensive care unit (ICU), according to algorithm-based priorities, stratified by ICU scarcity or nonscarcity setting. *p < 0.001; ^†^ *p* < 0.001

Physicians’ judgment of the appropriateness of ICU admission was correlated with algorithm-based priorities in both non-ICU bed scarcity and ICU bed scarcity settings (Fig. 1). The proportions of triage scenarios that were judged as appropriate for ICU admission in a non-ICU bed scarcity setting were 100 % (86 cases) for priority 1, 94 % (63 cases) for priority 2, 95.2 % (40 cases) for priority 3, and 67.1 % (104 cases) for priority 4 (*p* < 0.001). The proportions of triage scenarios that would be admitted to the last ICU bed were 83.7 % (72 cases) for priority 1, 61.2 % (41 cases) for priority 2, 45.2 % (19 cases) for priority 3, and 16.8 % (26 cases) for priority 4 (*p* < 0.001).

### Interrater reliability

In the interrater reliability analysis, overall agreement among algorithm-based priorities was 0.61 (95 % confidence interval [CI] 0.57–0.65), while overall agreement among physician-based priorities was 0.51 (95 % CI 0.47–0.55). Moreover, the median proportions (percentage) of agreement were 0.64 (IQR 0.59–0.70) for algorithm-based priorities and 0.49 (IQR 0.44–0.56) for physician-based priorities (*p* < 0.001). The average intraclass correlation coefficient for algorithm-based priorities was 0.94 (95 % CI 0.91–0.97), and for physician-based priorities it was 0.90 (95 % CI 0.85–0.94), both indicating good consistency in the prioritization process.

Agreement among individual components of the triage instrument is demonstrated in Additional file 1: Table S1. Individual questions demonstrated substantial interrater agreement (median κ values of 0.77, 0.74, and 0.84 for questions 1, 2, and 3, respectively), with the exception of question 4, with a median κ of 0.52.

### Correlation with clinical outcomes

There were 731 requests for ICU admission between September 2013 and December 2013. After excluding repeated requests, patients younger than 16 years of age, and requests for elective surgery, 603 patients were analyzed.

Hospital mortality was 37.8 % (224 patients), and 229 patients (38.0 %) were refused admission to the ICU. The algorithm-based priority distribution was as follows: 265 patients (45.5 %) were ascribed priority 1; 155 patients (26.6 %) were ascribed priority 2; 102 patients (17.5 %) were ascribed priority 3; and 61 patients (10.5 %) were ascribed priority 4.

**Fig. 2:**
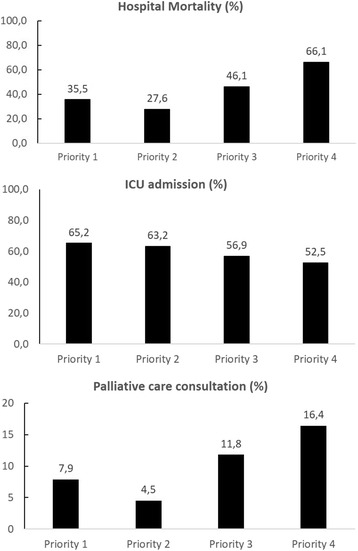
Association of priority categories with hospital mortality, intensive care unit (ICU) admission and palliative care consultation. Priorities 1, 2, 3, and 4 were statistically associated with hospital mortality (*p* < 0.001 by χ^2^ for trend), ICU admission (*p* = 0.035 by χ^2^ for trend), and palliative care consultation (*p* = 0.036 by χ^2^ for trend)

Hospital mortality was associated with priority level (Fig. 2). Priority 4 patients had a hospital mortality rate of 66.1 % (59 patients), while priorities 1, 2, and 3 patients had mortality rates of 35.5 % (92 patients), 27.6 % (42 patients), and 46.1 % (47 patients), respectively (*p* < 0.001 by χ^2^ for trend).

**Fig. 3:**
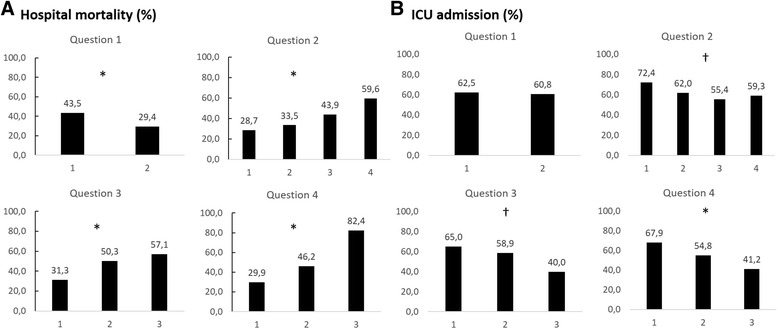
Association of individual questions with hospital mortality (**a**) and intensive care unit (ICU) admission (**b**). See supplementary material in Additional file 1 for number of patients in each group. *p < 0.001; ^†^ *p* < 0.05. Question 1: 1 = active intervention, 2 = monitoring. Question 2: 1 = no comorbidities, 2 = compensated comorbidities, 3 = decompensated comorbidities, 4 = advanced disease. Question 3: 1 = functionally independent, 2 = partially dependent, 3 = severely dependent. Question 4: 1 = probable survivor without severe disabilities, 2 = probable survivor with severe disabilities, 3 = probable nonsurvivor

The ICU admission rates were 65.2 % (172 patients) for priority 1 patients, 63.2 % (98 patients) for priority 2 patients, 56.9 % (58 patients) for priority 3 patients, and 52.5 % (32 patients) for priority 4 patients (*p* = 0.035 by χ^2^ for trend). Palliative care consultation was requested for 51 patients (8.5 %) in the cohort, with 21 (7.9 %) patients in priority 1, 7 (4.5 %) patients in priority 2, 12 (11.8 %) patients in priority 3, and 10 (16.4 %) patients in priority 4, as shown in Fig. 2 (*p* = 0.036 by χ^2^ for trend). Individual components of the tool were also evaluated and were associated with hospital mortality and ICU admission, as demonstrated in Fig. 3a and b and Additional file 1: Table S2.

## Discussion

This ICU triage algorithm has good reliability and validity, achieving construct validity by comparison of the results of the algorithm-based priorities with the reference standard, and by comparison with physicians’ judgment of appropriateness of ICU admission, in both nonscarcity and scarcity settings. Interrater reliability was substantial and was higher for the algorithm-based priorities than for physician-based priorities. The criterion (predictive) validity of this algorithm was further supported by correlation with surrogate clinical outcomes, such as ICU admission, palliative care consultation, and mortality. Moreover, when analyzing the individual components of the algorithm, it was found that each of these components contributed to the overall correlation.

There is growing consensus that clear and objective directives for ICU admission triage could benefit patient and resource allocation decision making [1, 6, 7]. Nevertheless, triage and risk stratification tools have not yet been routinely implemented in the ICU, other than in catastrophic settings [18, 19]. In part, this situation can be explained by the current absence of a validated triage tool for ICU admission, because the SCCM guidelines for ICU triage have never been formally evaluated, other than in small studies in which researchers examined specific priority strata [17, 20]. Moreover, it has been argued that existing physiological scores alone should not be used to predict outcomes [6]. Therefore, an alternative could be to use standardized subjective scores that combine objective data with the physician’s intuitive judgment of prognosis [21–23]. Such an instrument could be used to standardize and reduce biases that could adversely influence proper evaluation and decision making for ICU admission of individual patients.

This study has shown that this algorithm-based ICU triage decision-making tool has good interrater reliability, with a κ of 0.61, outperforming the interrater reliability of physicians’ prioritization. Because there are no ICU triage scales to use as benchmarks, this algorithm’s performance was compared with emergency triage scale performance reported in the literature. The authors of a systematic review of studies evaluating the Manchester Triage Score (MTS) [24] found a wide interrater agreement range. The ranges for unweighted and weighted κ statistics in these studies were 0.31–0.76 and 0.40–0.82, respectively. Of note, most studies were of low or moderate quality. The authors of another systematic review [25] found good reliability for the Canadian Triage and Acuity Scale and the Emergency Severity Index, with κ statistics ranging from 0.7 to 0.95, but found the MTS and the Australasian Triage Scale to be less reliable (κ statistics 0.3–0.6). Farrohknia et al. [26], in analyzing the current evidence on emergency triage scales, found no high-quality study available and also found a wide interrater agreement range, with κ values varying from 0.202 to 0.87.

Although comparable to rates for other published triage tools and higher than senior physicians’ agreement rates, these agreement rates are less than perfect. These agreement rates may be due to the inherent subjectivity associated with ICU triage [1], because disagreement on appropriateness of care may occur in up to 66 % of cases labeled as receiving inappropriate care [27]. To minimize this issue, we chose well-defined closed-end questions that have been shown to correlate with outcomes and to have high interrater reliability. The exception was question 4 (estimated prognosis), which was expected to be more intuitive and less reliable. This problem was mitigated by the fact that, in our algorithm, this specific question has less influence on the final priority. Question 4 would change only the ascribed priority from 3 to 4 in the case of a patient with advanced disease or severe functional dependence that would have been ascribed an intuitive estimate of no survival.

There are also controversies in the literature regarding the interpretation of κ values, and some suggest that the usual interpretation of 0.61–0.80 as substantial agreement [16] may be too lenient [28]. However, authors of many recent papers evaluating triage [24, 26, 29–32] and other scales such as the Glasgow Coma Scale [33] and the American Society of Anesthesiologists physical status classification [34] have used the more traditional interpretation of κ values and have shown similar agreement rates [24, 25, 34], which may help contextualize our results. It is possible that this more stringent approach was meant to be directed for more objective data, such as laboratory values [28]. Moreover, even when the more stringent approach was used, this tool’s performance was at least moderate and was probably better than the physicians’ intuitive priorities. In addition, as suggested by some authors [28], we have also reported other measures of reliability, such as percentage agreement and intraclass correlation coefficient, to help with interpretation of the results.

Validity, or the capacity of the tool to reflect the true triage priority, was evaluated. Because there is no established gold standard for prioritization or appropriateness of ICU, and in accordance with recommendations from the literature [9–11], the validity of this ICU admission triage algorithm was assessed by using a series of methods. In the context of the clinical vignettes, algorithm-based priorities demonstrated substantial agreement with the selected reference standard (two investigators with access to patients’ records), with a κ value of 0.72. In addition, the algorithm was shown to correlate with physicians’ judgment of appropriateness of ICU admission, especially in an ICU scarcity setting, demonstrating a linear relationship between prioritization and the proportion of scenarios that would be admitted to the last ICU bed. These results support the construct validity of the algorithm.

For further validation, this triage algorithm was compared with actual clinical outcomes in a subset of patients with requests for urgent ICU admission. The algorithm correlated with hospital mortality, palliative care consultation, and ICU admission, which supports its criterion (predictive) validity. However, although statistically significant, the difference in ICU admission rates between higher and lower priorities could be expected to be larger. Importantly, this observed difference is a reflection of current practice because the algorithm had no influence on ICU admission rates. Moreover, there is a perceived scarcity of ICU beds in our hospital, which is corroborated by the 30 % ICU refusal rate, so it is possible that this observed difference in admission rates may be due to a high rate of “inappropriate” admissions. Of note, despite the good correlation with outcomes in the retrospective cohort, the algorithm could not be properly evaluated regarding the association with benefit of ICU admission, defined as the difference in outcome with and without critical care [35].

This study has several strengths. It is one of the few to evaluate a triage tool for ICU admission and is, to our knowledge, the first to evaluate a triage algorithm for ICU admission in a resource-limited, noncatastrophic setting. Despite its single-center nature, in this study we evaluated a population of patients in a large academic tertiary hospital with 10 independent ICUs with different patient admission policies. Acknowledging the difficulty of establishing gold standards to ICU admission, we evaluated different surrogates covering different aspects of the process.

The study has several limitations, however. In the validation cohort, a short period of time was evaluated, and, due to the retrospective algorithm-based priority input, it was not possible to adjust for potential confounders and to ascertain all possible biases. Another important limitation is that in this study we evaluated the correlation of priority levels with outcomes, but the study was not designed to predict the impact of ICU admission on mortality in each priority stratum.

In addition, the clinical vignette methodology used, despite being based on real patients, may be less robust than real encounters with patients. Vignettes offer significant advantages, such as quantification of physician performance, ease of use, and low cost [29], and, as such, researchers in several studies have used case vignettes to study clinicians’ attitudes in both triage [29, 36–38] and nontriage settings [39–41]. Nevertheless, the vignette methodology differs from real patients in important ways because it summarizes and standardizes important clinical information and excludes the effect of external factors that could impact the decision-making process, such as time pressure and cognitive load [29]. It could be argued, for instance, that these vignettes’ characteristics could falsely increase the agreement rate in a subjective setting such as ICU triage. However, this is not consensual in the literature, because studies in which researchers have used case vignettes to evaluate decisions to withdraw support in critically ill patients [42] and patients with sepsis [43] and triage for ICU admission of typical ICU cases [44] have demonstrated wide disagreement in individual responses. Because the algorithm improved interrater agreement in the vignette methodology, it is possible to hypothesize that the algorithm would systematize the triage process and that this improvement could be even greater in the unstandardized setting of real patient encounters. Nonetheless, in this study we did not evaluate physicians’ performance with real patients, so we cannot be certain about the impact of the chosen methodology regarding the agreement rate.

Therefore, notwithstanding the good characteristics of this tool, this ICU admission triage algorithm may not be suitable for clinical practice yet, because, despite its correlations with outcomes, it was not properly evaluated for prediction of a differential benefit in each ICU priority stratum that would justify ICU admission of one stratum over the others. Further prospective studies with additional collected variables should be implemented to address the algorithm’s limitations.

## Conclusions

An algorithm-based prioritization system for ICU admission derived from the SCCM priority categories demonstrated good reliability and validity, with substantial interrater reliability and correlation with physician’s assessments and with the established reference standard. Algorithm-based priorities were also associated with clinical outcomes such as hospital mortality, palliative care consultation, and ICU admission, with lower priorities correlating with worse outcomes. However, at this stage, this algorithm could not be properly evaluated to predict the differential benefit of critical care for each priority stratum and may still not be ready for implementation. Further prospective studies that address these limitations are warranted.

## Key messages

- Algorithm-based priorities for ICU admission have good interrater reliability and correlate well with the judgment of appropriateness of ICU admission by a panel of experts.
- Algorithm-based priorities for ICU admission correlated with actual ICU admission, palliative care consultation, and hospital mortality in a retrospective cohort of patients for whom urgent ICU admission was requested.

## Additional file

Additional file 1: Supplementary material. (DOCX 68 kb)

## Abbreviations

ADL: activities of daily living
CI: confidence interval
HCFMUSP: Hospital das Clinicas da Faculdade de Medicina de Sao Paulo
ICU: intensive care unit
IQR: interquartile range
MTS: Manchester Triage Score
SCCM: Society of Critical Care Medicine
