# Supplementary material for: Development of an algorithm to aid triage decisions for intensive care unit admission: a clinical vignette and retrospective cohort study
Source: Crit Care. 2016 Apr 2;20:81. doi: 10.1186/s13054-016-1262-0 (PMC4818478; doi:10.1186/s13054-016-1262-0)
Supplement: Supplementary file 1 — Supplementary material. (DOCX 68 kb) [file 13054_2016_1262_MOESM1_ESM.docx]

Supplementary material

Ramos JGR, Peroni B, Daglius-Dias R; et al. Development of an instrument to aid triage decisions for intensive care unit admission: a clinical vignette and retrospective cohort study

**Table S1.** Correlations between individual components of the triage tool and hospital outcomes.

**Table S2.** Interrater reliability (quadratic weighted kappas) for individual components of the triage tool

**Appendix 1.** ICU triage tool algorithm framework

**Appendix 2.** English translation of vignettes samples

**eTable 1.** Correlations between individual components of the triage tool and hospital outcomes.

|  | **Hospital mortality** | **p** | **ICU admission** | **p** | |  |
| --- | --- | --- | --- | --- | --- | --- |
|  |  |  |  | |  | |
| **Question 1 (reason for ICU request)** |  | <0.001 |  | 0.795 | |  |
| Intervention, N(%) | 160/368 (43.5%) |  | 233/373 (62.5%) |  | |  |
| Monitoring, N (%) | 60/204 (29.4%) |  | 127/209 (60.8%) |  | |  |
|  |  |  |  |  | |  |
| **Question 2 (comorbidities)** |  | <0.001 |  | 0.03 | |  |
| No comorbidities, N(%) | 35/122 (28.7%) |  | 89/123 (72.4%) |  | |  |
| Compensated comorbidities, N(%) | 82/245 (33.5%) |  | 155/250 (62%) |  | |  |
| Decompensated comorbidities, N(%) | 76/173 (43.9%) |  | 97/175 (55.4%) |  | |  |
| Advanced disease, N(%) | 31/52 (59.6%) |  | 32/54 (59.3%) |  | |  |
|  |  |  |  |  | |  |
| **Question 3 (status performance)** |  | <0.001 |  | 0.008 | |  |
| Functionally independent | 126/402 (31.3%) |  | 266/409 (65%) |  | |  |
| Partially independent | 78/155 (50.3%) |  | 93/158 (58.9%) |  | |  |
| Severely dependent | 20/35 (57.1%) |  | 14/35 (40%) |  | |  |
|  |  |  |  |  | |  |
| **Question 4 (intuitive prognosis)** |  | <0.001 |  | <0.001 | |  |
| Survive without severe disabilities | 102/341 (29.9%) |  | 235/346 (67.9%) |  | |  |
| Surive with severe disabilities | 108/234 (46.2%) |  | 131/239 (54.8%) |  | |  |
| No survival | 14/17 (82.4%) |  | 7/17 (41.2%) |  | |  |

**eTable 2.** Interrater reliability (quadratic weighted kappas) for individual components of the triage tool

| **Question** | **Median** | **IQR range** | | **Min** | **Max** | **Mean** | **SD** |
| --- | --- | --- | --- | --- | --- | --- | --- |
| Question 1 | 0,77 | 0,70 | 0,83 | 0,60 | 0,92 | 0,76 | 0,08 |
| Question 2 | 0,74 | 0,70 | 0,81 | 0,54 | 0,91 | 0,75 | 0,09 |
| Question 3 | 0,84 | 0,78 | 0,93 | 0,58 | 0,98 | 0,84 | 0,10 |
| Question 4 | 0,52 | 0,36 | 0,60 | 0,11 | 0,80 | 0,48 | 0,16 |

**eAppendix 1.** ICU triage tool algorithm framework (Q1 = question 1; Q2 = question 2; Q3 = question 3; Q4 = question 4; P1 = priority 1)


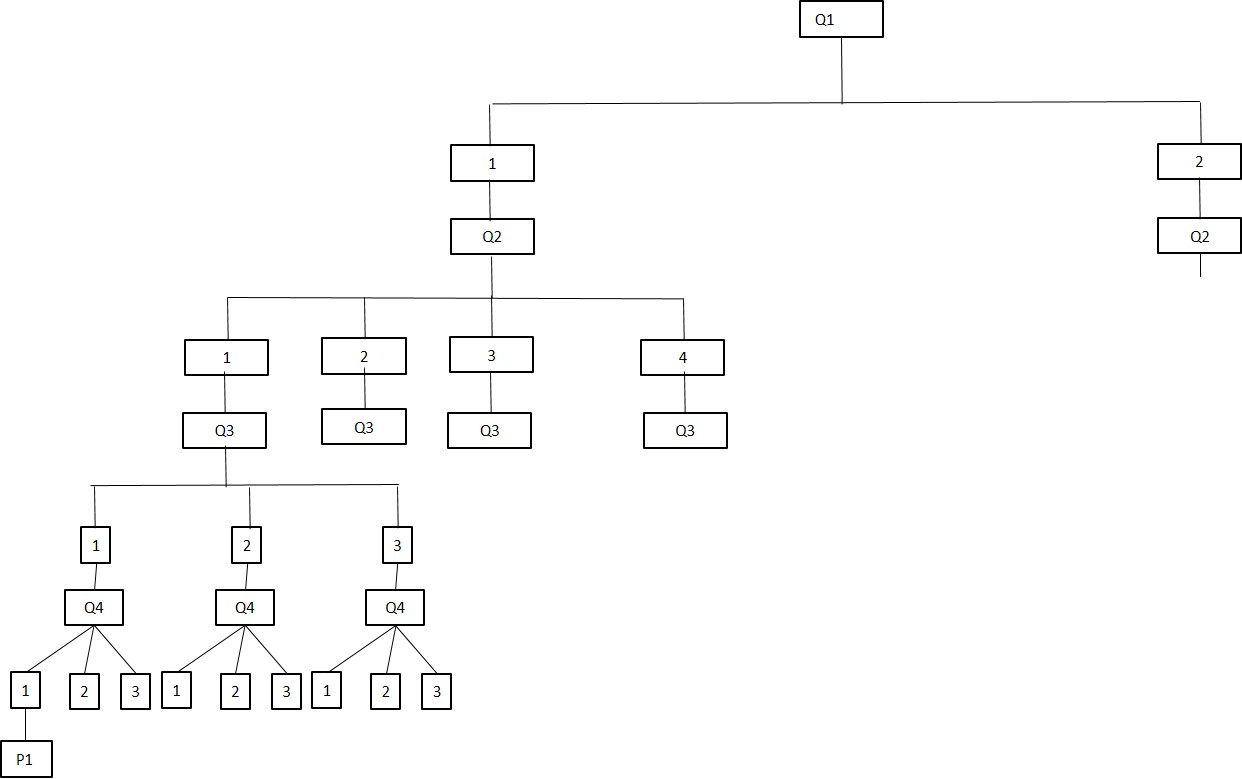


Example:

IF Question1 =1 AND Question2 =1 AND Question3 =1 AND Question4 =1 THEN

ASSIGN PRIORITY =1

**eAppendix 2.** English translation of vignettes samples

**Vignette sample Priority 1**

Female patient, 37 years-old, admitted in the hospital 3 days ago. Previous history of chronic kidney disease for 12 years was submitted to a kidney transplant (dead donor) 3 days ago, still in need of renal replacement therapy in the second post-operative day. Functionally independent for activities of daily living. Today, during dialysis, she developed sudden hypotension, associated with hemorrhagic drainage from surgical drains. Surgical team is aware and in the way. ICU admission was requested for somnolence, shock in use of vasopressors and need for urgent hemodialysis and surgical evaluation.

**Vignette sample Priority 2**

Female patient, 16 years-old, admitted in the hospital hours ago. Previous history of splenectomy secondary to trauma 5 years ago and a cesarean delivery 21 days ago. Functionally independent for activities of daily living. She was admitted with a history of fever and abdominal pain, with a diagnosis of endometritis after complimentary evaluation. She is awake, not hypotensive, mild tachycardic with remaining vital signs normal, but has an altered arterial lactate level. Treatment was begun in the emergency room and ICU admission was requested for severe sepsis, without need for artificial life support at this moment.

**Vignette sample Priority 3**

Male patient, 59 years, admitted to the hospital two weeks ago. Previous history of smoking and alcohol use, but has quit both 13 years ago. Previously independent for activities of daily living. He was admitted for evaluation of a cholestatic syndrome and weight loss of 13kg, with partial loss of functional independence. Workup revealed a diagnosis of cholangiocarcinoma. The tumor is not amenable to curative therapy or chemotherapy at this moment, so he was submitted to a palliative percutaneous transhepatic biliary drainage. After the procedure he developed hypotension with hemorrhagic drainage from surgical drains, with the need for vasopressors. ICU admission was requested for hemorrhagic shock in need for vasopressors.

**Vignette sample Priority 4**

Female patient, 68 years-old, admitted in the hospital two weeks ago. Previous history of bladder cancer with cystectomy 5 years ago and advanced Alzheimer’s disease. Completely dependent for activities of daily living. She was admitted in the hospital for treatment of an aspirative pneumonia and is evolving with worsening of the infection and has developed non-oliguric acute kidney injury and worsening of somnolence. ICU admission was requested for neurological and respiratory monitoring, without need for artificial life support at this moment.
